# Extending the diabetic retinopathy screening intervals in Singapore: methodology and preliminary findings of a cohort study

**DOI:** 10.1186/s12889-024-18287-2

**Published:** 2024-03-13

**Authors:** Amudha Aravindhan, Eva K. Fenwick, Aurora Wing Dan Chan, Ryan Eyn Kidd Man, Ngiap Chuan Tan, Wei Teen Wong, Wern Fern Soo, Shin Wei Lim, Sabrina Yi-Mei Wee, Charumathi Sabanayagam, Eric Finkelstein, Gavin Tan, Haslina Hamzah, Bibhas Chakraborty, Sanchalika Acharyya, Tai E. Shyong, Peter Scanlon, Tien Yin Wong, Ecosse L. Lamoureux

**Affiliations:** 1 Singapore Eye Research Institute and Singapore National Eye Centre, Singapore, Singapore; 2 Duke-NUS Medical School, Singapore, Singapore; 3 SingHealth Polyclinics, Singapore, Singapore; 4 Tan Tock Seng Hospital, Singapore, Singapore; 5 Saw Swee Hock School of Public Health, National University of Singapore and National University Health System, Singapore, Singapore; 6 Gloucestershire Hospitals NHS Foundation Trust, Gloucester, UK; 7 Tsinghua University, Beijing, China; 8 The University of Melbourne, Melbourne, Australia

**Keywords:** Diabetic retinopathy, Diabetic macular edema, Screening, Risk stratification

## Abstract

**Background:**

The Diabetic Retinopathy Extended Screening Study (DRESS) aims to develop and validate a new DR/diabetic macular edema (DME) risk stratification model in patients with Type 2 diabetes (DM) to identify low-risk groups who can be safely assigned to biennial or triennial screening intervals. We describe the study methodology, participants’ baseline characteristics, and preliminary DR progression rates at the first annual follow-up.

**Methods:**

DRESS is a 3-year ongoing longitudinal study of patients with T2DM and no or mild non-proliferative DR (NPDR, non-referable) who underwent teleophthalmic screening under the Singapore integrated Diabetic Retinopathy Programme (SiDRP) at four SingHealth Polyclinics. Patients with referable DR/DME (> mild NPDR) or ungradable fundus images were excluded. Sociodemographic, lifestyle, medical and clinical information was obtained from medical records and interviewer-administered questionnaires at baseline. These data are extracted from medical records at 12, 24 and 36 months post-enrollment. Baseline descriptive characteristics stratified by DR severity at baseline and rates of progression to referable DR at 12-month follow-up were calculated.

**Results:**

Of 5,840 eligible patients, 78.3% (*n* = 4,570, median [interquartile range [IQR] age 61.0 [55–67] years; 54.7% male; 68.0% Chinese) completed the baseline assessment. At baseline, 97.4% and 2.6% had none and mild NPDR (worse eye), respectively. Most participants had hypertension (79.2%) and dyslipidemia (92.8%); and almost half were obese (43.4%, BMI ≥ 27.5 kg/m^2^). Participants without DR (vs mild DR) reported shorter DM duration, and had lower haemoglobin A1c, triglycerides and urine albumin/creatinine ratio (all *p* < 0.05). To date, we have extracted 41.8% (*n* = 1909) of the 12-month follow-up data. Of these, 99.7% (*n* = 1,904) did not progress to referable DR. Those who progressed to referable DR status (0.3%) had no DR at baseline.

**Conclusions:**

In our prospective study of patients with T2DM and non-referable DR attending polyclinics, we found extremely low annual DR progression rates. These preliminary results suggest that extending screening intervals beyond 12 months may be viable and safe for most participants, although our 3-year follow up data are needed to substantiate this claim and develop the risk stratification model to identify low-risk patients with T2DM who can be assigned biennial or triennial screening intervals.

**Supplementary Information:**

The online version contains supplementary material available at 10.1186/s12889-024-18287-2.

## Introduction

Early detection and timely treatment can reduce incidence and progression of diabetic retinopathy (DR), a major cause of vision loss and blindness [1–4]. Guidelines for regular retinal examinations in people with diabetes (DM) are available in many developed countries [5–8]. In Singapore, the Ministry of Health recommends annual screening for patients with non-referable DR (i.e. no or mild non-proliferative DR-NPDR, and no DME- diabetic macular edema) [9], which is conducted at primary care via a tele-ophthalmology platform, the Singapore integrated Diabetic Retinopathy Programme (SiDRP). While SiDRP is accurate, and time- and operationally-effective, repeated yearly checks are expensive and burdensome, especially given that declines in incidence and progression rates of vision threatening DR (VTDR) have been demonstrated [10–13]. With the future prevalence and economic burden of DM expected to soar in Singapore [14–16], it is imperative that screening policies for DM-related complications are cost-effective and sustainable.

Studies conducted elsewhere have concluded that the interval between diabetic eye screening visits could be safely extended beyond 12 months [17] with high adherence rates [18] and no undue delays in treatment for referable DR in patients with diabetes without DR [19–21], Moreover, extending the screening intervals beyond one year has been found to be cost-effective [22–24]. However, it is uncertain if extended screening intervals are appropriate for a multi-ethnic Asian population with DM due to differences in healthcare systems, compliance to glucose control, and prevalence, risk factors and burden of DM and DR [25–28]. Indeed, a systematic review in 2016 by Taylor-Phillips and colleagues found insufficient evidence to recommend extending the screening interval beyond one year [29]. Moreover, most studies have analyzed retrospective cohort data [18, 30] or lacked information on key risk factors for DR progression [31–33], such as duration of DM, insulin use, glycaemic and lipids profile, blood pressure (BP), and vascular complications [34]. Such information is crucial to identify low-risk patients with DM in whom the screening interval can be extended without the risk of VTDR developing before the next screening visit.

We have implemented a large, prospective study – the Diabetic Retinopathy Extended Screening Study (DRESS)—to develop and validate a DR/DME risk stratification model using eye screening, sociodemographic, lifestyle, medical and routinely collected clinical information from SiDRP patients with type 2 DM (T2DM), to guide assignment of low-risk patients to biennial or triennial DR screening intervals. DRESS has three specific aims: (i) to develop a DR/DME risk estimation algorithm using eye screening results and risk factors of progression, and identify which subgroups of patients with T2DM may be offered a biennial or triennial rescreen, as opposed to annual, by determining the 2- and 3-year progression rates of yearly non-referable to referable DR/DME patients; (ii) to externally validate this algorithm in an independent sample of patients with T2DM using similar grading protocol and referral criteria; (iii) to estimate the net cost implications and incremental cost-effectiveness ratios for biennial and triennial screening of patients with T2DM at low-risk of progression versus annual screening as mandated in current clinical management guidelines. In this paper, we describe the protocol and preliminary findings, including baseline sociodemographic and clinical characteristics of participants and the rate of progression to referable DR at the first annual follow-up.

## Methods

The study is being implemented at four of the nine SingHealth Polyclinic (SHP) locations (Supplementary materials), namely Bedok and Pasir Ris (East of Singapore), and Outram and Bukit Merah (South). Recruitment commenced in December 2017, March 2018, October 2018 and January 2019 at Bedok, Outram, Pasir Ris and Bukit Merah, respectively, and ended at all sites in May 2021. The 12-, 24-, and 36-month follow-up data collection is currently ongoing.

### Study population and design

Patients with T2DM (primary or secondary diagnosis in the medical records) who (a) underwent DR screening at SiDRP at one of the four primary care polyclinics, with non-referable DR/DME in both eyes; and b) on the day of study enrolment received a referral for an annual rescreen, satisfied DRESS inclusion criteria. Eligible participants were also Singaporean citizens or permanent residents of Chinese, Malay, Indian, or Eurasian ethnicity; aged ≥ 21 years; and free of significant hearing impairment that could interfere with study enrolment and data collection, and cognitive impairment as assessed using six-item Cognitive Impairment Test (6-CIT) [35]. Patients with type 1 DM (T1DM); referable DR/DME; < 12 months rescreen referral for non-referable DR/DME; or ungradable fundus images at baseline, were ineligible.

**Fig. 1:**
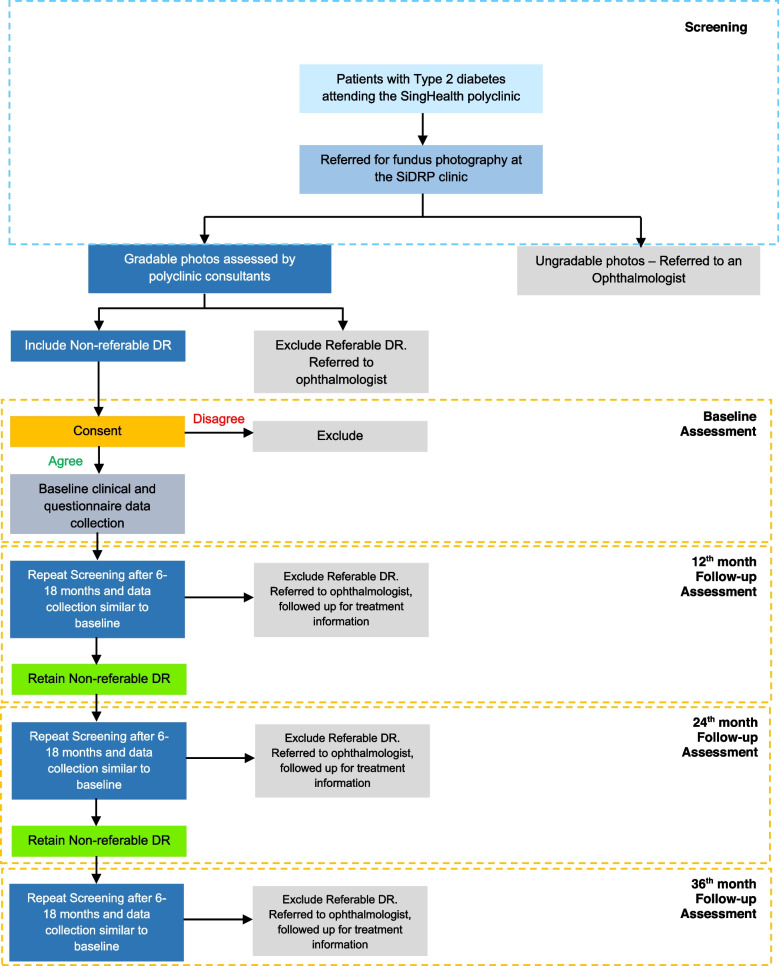
DRESS Baseline and Follow-up Protocol

Each individual completed a baseline assessment on the day of enrolment, and will be followed up at their 12-, 24- and 36-month rescreen visits (Fig. 1), with a window period of ± 6 months for each follow-up point. Therefore, follow-up screening episodes will be considered to be at 1 year if they fall within 7–18 months of the first visit, 2 years if within 19–30 months, and so forth. Those who progress to referable disease at any rescreen visits post-baseline recruitment will be referred for appropriate tertiary treatment and will thereafter be followed up for obtaining information about the treatment they received.

Ethics approval was obtained from the SingHealth Centralized Institutional Review Board (CIRB, Reference #2016/2439), and the DRESS protocol adheres to the principles of the Declaration of Helsinki. Prior to data collection, participants signed a written informed consent form in their preferred language (English, Mandarin, Malay or Tamil).

### Recruitment

#### Screening for eligibility and enrolment

On the day preceding the SiDRP screening, potentially eligible individuals were sourced from the electronic medical records (EMR) by a trained clinical research coordinator (CRC) at the SHP. On the DR screening visit, eligible patients from the initial list were approached by the CRC and, if they passed the hearing and cognitive assessments, were invited to participate. Screen fails were also identified during the face-to-face assessment.

There were three outcomes at the point of recruitment: the patient agreed to participate, declined, or provided no definitive response. De-identified data (age and gender) of patients who refused participation were collected.

### Assessment and referral procedure in SiDRP

SiDRP assessment took place at baseline, and is currently ongoing at 12-, 24- and 36- months rescreening visits.

#### Assessment of DR or DME

Following dilation, 2-field photographs (optic-disc and macula) using a non-mydriatic 45º fundus camera are obtained for both eyes by an ophthalmic nurse. Images are transferred to the Singapore Ocular Grading Centre and graded within 1 h using a standardized protocol by trained graders masked to the patients’ characteristics (Supplementary materials). Patients are given immediate feedback on the screening test at the same polyclinic visit and, if necessary, receive an appointment to a tertiary eye care facility for further treatment.

#### Referral Criteria for DR

**Fig. 2:**
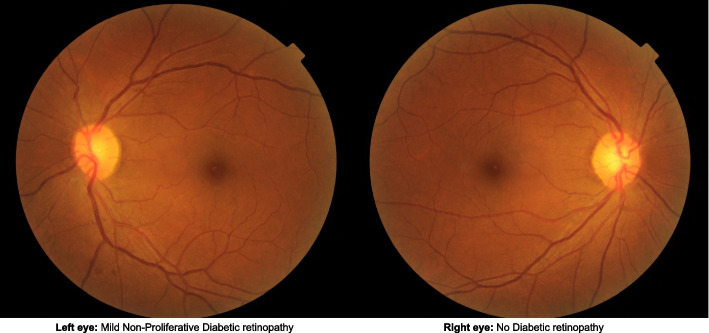
Examples of two-field fundus images of a DRESS participant with non-referable diabetic retinopathy

Patients are considered to have DR if any of the following grading features are present in any eye: microaneurysms (MA), hemorrhages, cotton wool spots, intra-retinal microvascular abnormalities, hard exudates, venous beading, and new vessels. The DR level for each patient is derived by concatenating the level for the two eyes, giving the eye with the higher level greater weighting. Based on the feature grading parameters, DR is classified as none; mild; moderate; severe NPDR; and proliferative diabetic retinopathy (PDR). Referable DR is defined in the SiDRP protocol as moderate NPDR or worse. Those with none or mild NPDR (Fig. 2) are advised to have an annual rescreen at the polyclinic (Supplementary Table 1). For those with referable DR, the referral criteria will vary between one week (PDR) to three months (moderate NPDR) at a tertiary eye care centre.

Images are considered ungradable if retinal vessels are not visible within 1-disc diameter of the centre of the fovea, fine vessels are not visible across the surface of the optic disc, severe obscuration of 1 or more quadrant or of macula by artifacts, or no view of fundus up to 1 disc diameter beyond vascular arcade. If the image of one eye is ungradable, the severity levels will be considered to be equivalent to that of the other eye.

#### Referral Criteria for DME

No DME, with an annual rescreen recommendation (Supplementary Table 2), is defined as no DR signs within the macula and/or any MAs/dot hemorrhage (DH) /blot hemorrhage (BH) within the outer zone with visual acuity (VA) better than 6/12. These patients were eligible to participate in the DRESS.

### Study assessments and data extraction

During the baseline assessment, demographic, socio-economic and lifestyle information was obtained via face-to-face, interviewer-administered questionnaires (Supplementary materials). Height and weight were obtained from participants medical records. BP was assessed using a digital automatic BP monitor [36] if not available in the case notes. Participants’ HbA1c, lipids [low/high density lipoprotein cholesterol (LDL/HDL), triglycerides (TG), total cholesterol (TC)], serum creatinine, urine spot albumin: creatinine ratio (ACR), and estimated glomerular filtration rate (eGFR) were collected from case notes if assessed ≤ 12 months ago. Otherwise, a venous blood sample and mid-stream urine sample were collected for same-day analysis. Distance VA, duration of DM, and medical history and medication were collected from case notes. The 12, 24 and 36 month follow-up sociodemographic, lifestyle, medical, ocular and clinical data are currently being extracted from medical records, and adhere to data quality assurance and control standards (Supplementary materials). Additionally, those who progress to referable disease at any rescreen visits post-baseline will be followed up for obtaining information about the treatment they received.

### Sample size

The sample size to develop a DR/DME risk stratification model was based on estimates of progression rates and probabilities of referable DR/DME from the literature [37]. In the study by Looker and colleagues, the 1-year progression rate for those with no DR vs. mild DR was 0.3% vs. 5.8% (relative risk (RR) = 19), respectively, and corresponding estimates for 2-year progression rate were 1.1% vs. 12.2% (RR = 11) [38]. Assuming that 20% of our patients have mild DR [39], and a 15% progression rate among those with no DR, a sample of 1,652 and 413 patients with no DR and mild DR respectively, would allow detection of relative risk as small as 1.41 with 80% power and α = 0.05 for a two-sided test. Overall, DRESS required a sample size of 2,065 to develop the risk estimation algorithm and an additional 2,065 participants for validation. Assuming a 10% drop out rate in the consecutive annual rescreening visits, the final estimated sample size needed was ~ 4,590 patients.

### Analytical plan

We will divide the population into ‘derivation’ and ‘validation’ data sets by randomly selecting 50% of the enrolled patients (*n* = 2,295) for the model derivation data set and reserving the other 50% for model validation (*n* = 2,295).

For *Aim 1*, we will use prospective data from the derivation cohort (*n*= 2,295) to calculate progression rates at 12-, 24- and 36-month rescreening visits by dividing the number of persons with referable DR or DME by the number of participants enrolled at baseline stratified by severity of DR. The progression in DR severity will be calculated by dividing the number of persons who have progressed to referable status from baseline by the number of persons at baseline. The converse will be applied to compute regression rates for DR [11]. The probability of progression to referable DR or DME will be computed using Hidden Markov Models (HMM) [37], where three observed states of no DR; mild NPDR; and referable-DR will be modelled. For DME, we will have two observed states, namely annual rescreen and referable DME. The model will be fitted to the data and the effect of individual level covariates (including age, gender, ethnicity, duration of DM, diabetic complications, poor glycemic control [HbA1c ≥ 7%], lipids, body mass index [BMI], BP and eGFR at baseline) will be examined on the progression probability between states of DR and DME severity. The model-based probabilities of observing a progression to referable DR/DME in the ensuing 1, 2 and 3 year periods according to variables shown to influence progression intensities will be reported. We will develop separate risk estimation models for DR (including one for progression to PDR alone) and DME, and the different ethnic groups. A prognostic model to predict the risk of developing referable DR will be developed based on methods adopted by Aspelund and associates [40], and the probability of not developing referable DR within a specified time interval for a subject with non-referable DR at baseline will be derived using a parametric Weibull proportional hazard model. The model with the best fit will be selected using Akaike information criterion and by plotting the fitted survival curve overlaid on the Kaplan–Meier survival curve.

For *Aim 2*, we will validate the DR and DME risk estimation models developed in Aim 1 on our validation cohort (*n*= 2,295). The predictive adequacy of the models developed in Aim 1 will be measured by calculating appropriate statistics for time to event data, which are equivalent to discrimination and calibration statistics for predictive models based on logistic regression [41].

For *Aim 3*, we will quantify the incremental cost-effectiveness of biennial or triennial screening intervals relative to annual screening for low risk intervals based on the risk stratification tool developed in Aim 2 and a Markov model that captures the short and longer term costs and outcomes for the cohort. We will rank the interventions in terms of increasing costs and quantify the incremental cost-effectiveness of each screening modality relative to its next most costly alterantive. Costs will focus on the payer perspective and include all screening and subsequent treatment costs, including costs for false positives. Effectiveness will focus on quality adjusted life years gained (QALYs) based on well defined relationships between visual actuity and QALYs. Both one way and probabilistic sensivity analyses will be conducted.

### Preliminary statistical analyses

Preliminary analyses were performed using Stata/SE, version 15 (StataCorp, College Station, Texas). First, we compared the age and gender of participants and non-participants (those who refused) using a t-test or chi-square test. Second, we calculated descriptive statistics (mean and standard deviation [SD] or median and interquartile range [IQR], counts and percentages) for participant sociodemographic, medical and clinical characteristics at baseline. Third, we compared the characteristics of participants with no DR and mild NPDR at baseline using the Mann–Whitney U-test, or chi-square and Fisher exact test. Fourth, we calculated the proportions of those who progressed to referable DR at the first annual follow-up post enrollment.

## Results

### Screening and recruitment

**Fig. 3:**
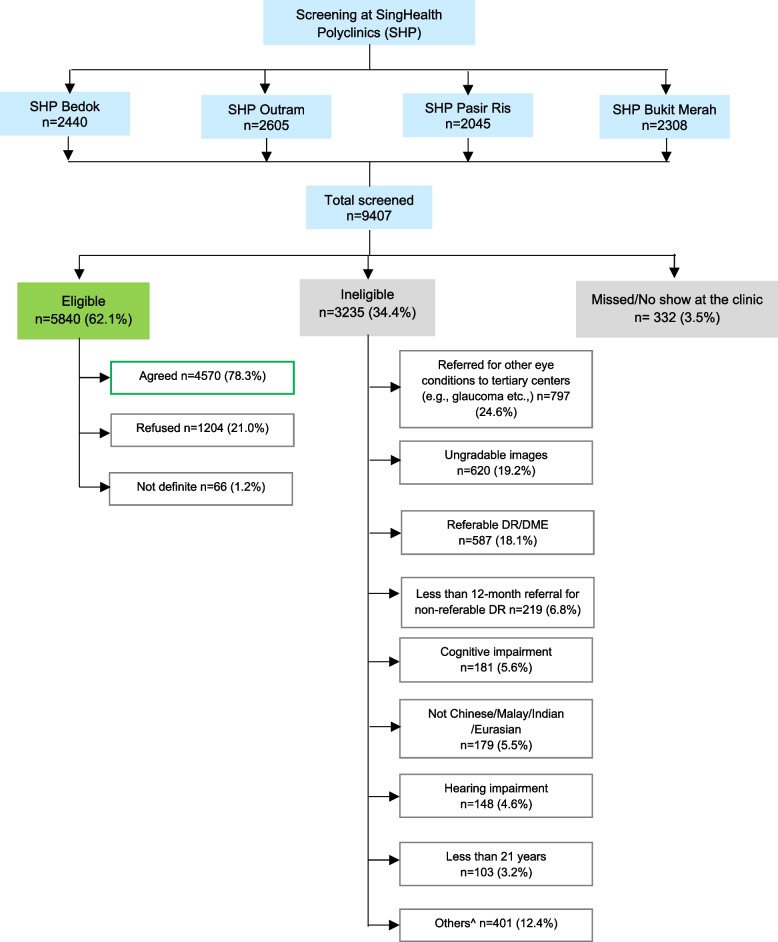
DRESS Screening and Recruitment

A total of 9,407 patients were screened from the four polyclinic sites. Of the 5,840 (62.1%) eligible patients, 4,570 (78.3%, response rate) agreed to participate, and 1,204 (21%) and 66 (1.1%) refused, or were undecided, respectively (Fig. 3). Reasons for refusal included not interested (*n* = 553, 46.0%), time commitment too great (*n* = 195, 16.2%), multiple study follow-up assessments (*n* = 178, 14.8%), need to share confidential information (*n* = 77, 6.4%), blood and urine sample collection requirement (*n* = 84, 7.0%), and other reasons (e.g., do not believe in research, caregiver does not approve etc., *n* = 103, 8.6%). Compared to participants, non-participants were older (mean ± SD 60.3 ± 9.6 vs. 62.8 ± 9.7) and more likely to be female (45.3% vs. 52.6%, both *p* < 0.001).

Of the 9,407 patients screened, 3,235 (34.4%) were ineligible of which a quarter (*n* = 797, 24.6%) were referred to tertiary centres due to other eye conditions (e.g., glaucoma). Moreover, almost one-fifth (*n* = 620, 19.2%) had ungradable fundus images, 587 (18.1%) had referable DR/DME and 219 (6.8%) received a < 12 months rescreen referral for non-referable DR/DME (Fig. 3).

### Sociodemographic, medical and clinical characteristics

Overall, the median [IQR] age of the 4,570 participants was 61 [55-67] years; and 54.7% were male. The ethnic composition of participants was 68% Chinese, 22% Malay, 9.6% Indian and 0.5% Eurasian. The median [IQR] self-reported duration of DM was 6 [3–10] years, and 6.5% (*n* = 299) were on insulin. Most participants had hypertension (79.2%) and dyslipidaemia (92.8%), and almost half were obese (43.4%, defined as BMI ≥ 27.5 kg/m^2^ using Asian-specific cut-offs [42]).

Baseline participants had a median HbA1c; and systolic and diastolic BP values of 6.8% [IQR 6.3–7.4], 130.5 [IQR 121.5–138.0] mmHg, and 70.0 [IQR 64.5–77.0] mmHg, respectively. The median LDL cholesterol and eGFR was 2.1 [IQR 1.7–2.5]mmol/L and 87.8 [IQR 73.5–97.9] mL/min/1.73m^2^, respectively.

Baseline characteristics are also presented by age, gender and diabetes duration (Supplementary Table 3–5).

The percentage of missing values was 1% or less in all variables considered, except income (27.1%) and urine albumin/creatinine (26.6%) (Supplementary Table 6).

### Preliminary findings on non-referable DR and its progression

Compared to those with mild NPDR (2.6%), those with no DR (97.4%) reported shorter DM duration, and had lower HbA1c, TG (all *P* < 0.05) and urine albumin/creatinine/ ratio (*P* < 0.001), and higher HDL cholesterol (*P* < 0.05; Table 1). Furthermore, they were less likely to have had a stroke and be on oral anti-diabetic medications (all *P* < 0.05).

**Table 1:** Characteristics of Type 2 diabetes patients with no diabetic retinopathy and mild non-proliferative DR at baseline (*n* = 4570)

Characteristics	No DR (*n* = 4449)	Mild NPDR (*n* = 121)	*P*
Age (years), median (IQR)	61.0 (55.0–67.0)	60.0 (55.0–67.0)	0.295
Male gender, n (%)	2429 (54.6)	70 (57.9)	0.478
Diabetes duration (years), median (IQR)	6.0 (3.0–10.0)	8.0 (3.0–11.0)	0.037
Ethnicity, n (%)			0.843
Chinese	3028 (68.1)	79 (65.3)	
Malay	971 (21.8)	29 (24.0)	
Indian	427 (9.6)	13 (10.7)	
Eurasian	23 (0.5)	0 (0.0)	
Educational attainment, n (%)			0.722
Primary or lower	1005 (22.6)	29 (24.0)	
Secondary or above	3443 (77.4)	92 (76.0)	
Housing type, n (%)			0.085
Public	3921 (88.3)	113 (93.4)	
Private	519 (11.7)	8 (6.6)	
Monthly Household Income, n (%)			0.679
< $2000	1164 (36.0)	34 (34.0)	
$2000 and above	2068 (64.0)	66 (66.0)	
Occupation, n (%)			0.488
Unemployed	155 (3.5)	7 (5.8)	
Housewife	499 (11.2)	13 (10.7)	
Retired	1113 (25.0)	26 (21.5)	
Working	2682 (60.3)	75 (62.0)	
Marital status, n (%)			0.287
Single or never married	564 (12.7)	14 (11.6)	
Married	3359 (75.5)	87 (71.9)	
Separated, divorced or widowed	526 (11.8)	20 (16.5)	
Polyclinic Location, n (%)			0.129
Bedok	1203 (27.0)	37(30.6)	
Bukit Merah	911 (20.5)	23 (19.0)	
Outram	1113 (25.0)	38 (31.4)	
Pasir Ris	1222(27.5)	23 (19.0)	
Lives alone (yes), n (%)	365 (8.2)	14 (11.6)	0.185
Smoking status, n (%)			0.335
Never	3409 (76.6)	88 (72.7)	
Past	471 (10.6)	12 (9.9)	
Current	569 (12.8)	21 (17.4)	
Alcohol use, n (%)			0.445
Never	3390 (76.2)	96 (79.3)	
Past	315 (7.0)	5 (4.1)	
Current	744 (16.7)	20 (16.5)	
BMI (kg/m2), median (IQR)	26.7 (24.1–30.2)	26.9 (24.1–30.5)	0.559
BMI categories (kg/m2)			0.898
< 18.5	34 (0.8)	1 (0.8)	
18.5—23.0	657 (14.9)	16 (13.2)	
23.0—27.5	1814 (41.0)	49 (40.5)	
≥ 27.5	1919 (43.4)	55 (45.4)	
Blood pressure (mmHg), median (IQR)			
Systolic	130.0 (121.5–138.0)	133.0 (124–139.0)	0.054
Diastolic	70.0 (64.5–77.0)	69.0 (64.0–77.0)	0.309
Hypertension^b^, n (%)	3520 (79.1)	101 (83.5)	0.244
Dyslipidaemia^c^, n (%)	4128 (92.8)	111 (91.7)	0.660
History of cardiovascular disease, n (%)			
Coronary artery disease (yes)	506 (11.4)	18 (14.9)	0.242
Stroke	179 (4.0)	10 (8.3)	0.021
Kidney disease, n (%)	620 (14.0)	19 (15.7)	0.580
Diabetes treatment, n (%)			
Insulin	288 (6.5)	11 (9.1)	0.251
Oral anti-diabetic medications	3395 (76.3)	105 (86.8)	0.007
PVA (Better eye)			0.683
None (LogMAR ≤ 0.3)	4228 (95.1)	116 (95.9)	
Mild (LogMAR > 0.3)	220 (4.9)	5 (4.1)	
HbA1c (%),median (IQR)	6.8 (6.3–7.4)	7.0 (6.5–8.0)	0.004
Lipids, median (IQR)			
Total cholesterol (mmol/L)	4.0 (3.57–4.6)	3.9 (3.4–4.6)	0.146
Triglycerides (mmol/L)	1.4 (1.0–1.8)	1.5 (1.2–2.1)	0.004
HDL cholesterol (mmol/L)	1.3 (1.1–1.5)	1.2 (1.0–1.4)	0.001
LDL cholesterol (mmol/L)	2.1 (1.7–2.5)	2.0 (1.6–2.3)	0.046
Serum creatinine (umol/L), median (IQR)	75.0 (62.0–89.0)	76.0 (64.0–90.0)	0.413
eGFR^a^ (ml/min/1.72m2), median (IQR)	88.0 (73.5–97.9)	88.0 (72.2–98.3)	0.920
Urine albumin/Creatinine (mg/mmol), median (IQR)	2.0 (1.1–4.7)	3.4 (2.0–10.1)	< 0.001

*BMI* Body Mass Index, *DR* Diabetic Retinopathy, *NPDR* Non-proliferative diabetic retinopathy, *PVA* Presenting Visual Acuity, *HbA1c* Haemoglobin A1c, *IQR* Interquartlie range, *HDL* High Density Lipoprotein, *LDL* Low Density Lipoprotein, *eGFR* Estimated Glomerular Filtration Rate

^a^eGFR was calculated based on the CKD-EPI formula

^b^Hypertension – primary or secondary clinical diagnosis of hypertension in the medical records

^c^Dyslipidemia – primary or secondary clinical diagnosis of dyslipidemia in the medical records

Of the 4,570 study participants enrolled, we have extracted 41.8% (*N* = 1909) of the first annual follow up data to date. In this preliminary follow-up, on average, participants attended their screening 12.7 ± 1.9 months after baseline enrollment. Of these, 99.7% (*n* = 1,904) did not progress to referable DR (no DR [96.5%] and mild NPDR [3.5%]). Those who progressed to referable status (0.3%; moderate [*n* = 4] and severe [*n* = 1] NPDR) had no baseline DR but all were dyslipidaemic, and 60% had hypertension and obesity. The 5 participants who progressed to referrable DR had a median DM duration of 13.0 years, with median HbA1c, LDL cholesterol and eGFR levels of 7.5%, 2.7 mmol/L, and 96.8 mL/min/1.73m^2^, respectively, at baseline. Although there was no change in DR severity for most participants (94.6%), 3.1% (*n* = 60) and 2.3% (*n* = 43) participants progressed and regressed by at least one grade, respectively. Those who progressed by one step had no baseline DR, and most were Chinese (76.7%) and had hypertension (70.0%) and dyslipidemia (91.7%) at baseline.

## Discussion

DRESS will be the first prospective cohort study to develop and validate a DR/DME risk stratification model to guide assignment of low-risk patients to biennial or triennial DR screening intervals using a large, well-characterized dataset from a multi-ethnic Asian population with T2DM attending a national eye screening program. Almost 4 in 5 of those eligible were recruited and have completed the baseline assessment. Although most patients had no DR (97.4% vs 2.6% with mild NPDR) at baseline, the prevalence of hypertension, dyslipidemia, and obesity was high. Despite this, our preliminary findings based on over 40% of the sample extracted from the ongoing 12-month follow-up indicate that > 99% of patients with non-referable DR at baseline did not progress to referable status. Study completion in November 2024 will allow us to develop an evidence-based algorithm to guide primary healthcare providers in Singapore to prescribe biennial and triennial screening for low-risk patients with T2DM; and inform policymakers and researchers about the cost-effectiveness of this approach.

The prevalence of mild NPDR in our baseline screening was lower (2.6%) than that reported in the UK (25.9%) [43], US (14.9%) [44], Denmark (6.6%) [45], Brunei (7%) [46], Indonesia (9.4%) [47], China (20.4%) [48–50] and a previous study in Singapore (17.7%) [51], but similar to a recent population-based study conducted in urban China (2.1%) [52]. These variations could be attritubed to differences in grading definitions of mild NPDR [39], study population (e.g., community-based, primary clinic-based) [51], ethnicity [26], urbanization [53], duration of DM and level of DM control [49], health literacy [54], and preventative measures [4]. We investigated the overall prevalence of mild NPDR in the four participating Polyclinics from 2018 to 2020 (during which > 70% of our data were collected) and compared these with DRESS. The prevalence reported in DRESS (2.6%) was similar to overall 2018 (3.2%), 2019 (3.4%) and 2020 (3.5%) levels, albeit still somewhat lower. Study selection biases, including the higher proportion of Chinese [51] and younger (i.e. proxy for shorter duration of DM) people and study sites may explain the approximately -0.6% (e.g. 3.2% vs. 2.6%) prevalence of mild DR in DRESS compared to the general Polyclinic population.

The very low incidence of referable DR (0.3%) observed in our cohort of patients with no DR at 12 months is similar to that reported in the Liverpool Diabetic Eye Study [55], and in large observational studies conducted in seven DM retinal screening programs across the UK and Scotland. The Liverpool cohort followed up 3,743 patients with T2DM without DR for 6 years and found that the incidence of VTDR was low in the first year (0.3%), rising to 1.8% in the fifth year. Similarly, in the UK study, Leese and colleagues found that the expected proportion of patients without DR at baseline to progress to referable disease at 1 year ranged between 0.1 to 0.6% [56]. In a retrospective study of 300,101 patients with T2DM and no DR who attended the Scottish Diabetic Retinopathy Screening Program at baseline, Looker and associates found that only 0.6% progressed to referable DR at the next annual assessment [38]. Notwithstanding differences in study design (e.g. definitions of DR, duration of follow-up, and statistical methods), evidence from our study and others support the recommendation that screening intervals could be safely extended beyond 12 months in T2DM patients with no DR.

Interestingly, the 5 patients who progressed to referable DR did not have DR at baseline. However, they were dyslipidaemic and most had hypertension, and had median DM duration of 13 years, and median HbA1c and eGFR levels of 7.5% and 96.8 mL/min/1.73m^2^, respectively, at baseline. Several studies conducted in Asian countries have reported presence of hypertension and dyslipidaemia, long DM duration, and high HbA1c and eGFR levels as risk factors of incidence and progression of DR [57, 58], and this likely explains the progression from no DR to referable DR in the 5 patients in our study. Importantly, our current findings on the progression to referable DR at 12 months (0.3%) is based on only two-fifths of our 1st annual follow-up data. As such, we require the complete 12-month follow-up data to have a full understanding of the progression rates and patient baseline characteristics associated with progression to referable DR.

Our Singaporean primary care patients had reasonably good metabolic and BP control and short DM duration (< 10 years), particularly among those with no DR, and this could have contributed to the low DR progression rates seen in our preliminary 12 month findings. Indeed, several studies have suggested that it may be possible to reduce the number of screening visits to once every 2–5 years in patients with a T2DM duration of < 10 years with good glycaemic and BP control [20, 32, 59, 60]. As such, our early findings suggest that annual screening in Singapore may not be necessary for patients with T2DM, no DR and good metabolic control.

Being a prospective and pragmatic study means that our future revised DR screening protocol will need to adapt to healthcare technology innovations, such as AI models, which have shown excellent results in detecting any DR from retinal images, compared to human assessors [61]. Indeed, the Singapore Eye LEsioN Analyser (SELENA +), a deep learning system developed and validated in Singapore, will be adopted at the four DRESS sites for routine eye screening in 2024. The SELENA + screening model will include an automated triage followed by secondary human assessment of all cases flagged as having referable DR [62, 63]. Recommendations will be provided for annual rescreening at polyclinics and referrals for tertiary eye care for non-referable and referable DR, respectively [64]. This semi-automated system is quicker [65] and less expensive [64] compared to the current full human assessment system used in SiDRP. However, cost-effectiveness of a population-based screening program also depends on the frequency of retinal examinations and imaging. Therefore, our DR risk stratification models, which will allow extension of the screening interval beyond one year for low-risk patients, could complement the SELENA + model to further improve cost-effectiveness. Nevertheless, further evaluation of the clinical- and cost-effectiveness of using our DR risk stratification algorithm within SELENA + is required.

Strengths of the DRESS protocol include its (a) real-world data, (b) large sample size representative of three major Asian ethnic groups (Chinese, Malay and Indian), all at increased risk of DM-related complications compared to Caucasians [66]; (c) prospective, long-term follow-up (~ 20,000 screening visits) design, which not only explores the progression of DR but allows the collection of routine clinical data crucial to the development of an accurate risk algorithm to identify low-risk progression patients [34, 67]; (d) good response rate; and (e) systematic grading of fundus images, based on a standardized protocol by a centralized team of trained graders at SiDRP [32, 67]. Potential limitations include: (a) selection bias due to exclusion of individuals with significant hearing and cognitive impairment, and restriction of recruitment to only patients with T2DM undergoing DR screening at polyclinics; (b) high non-participation among older patients and females with T2DM; (c) non-applicability of our algorithm to adults with T1DM; d) lack of data on progression after referral to tertiary centres, meaning that our algorithm will not be sensitive to high-risk primary care T2DM patients; and e) non-use of gold standard techniques such as optical coherence tomography for detection of DME. Additionally, there was no study participant enrolment between February 2020 to June 2020 due to the the Coronavirus Disease (COVID-19) pandemic and related restrictions (e.g., restriction to face-to-face research activities) (https://www.moh.gov.sg/news-highlights/details/risk-assessment-raised-to-dorscon-orange), an unprecedented period during which population health research implementation was affected globally [68, 69]. It is possible that we may have missed recruiting potentially eligible patients in our study during this period.

The future focus of DRESS will be on validating the risk stratification models in other healthcare clusters in Singapore; and testing the clinical outcomes, safety, and sustainability of the DR/DME risk stratification models in a real-world diabetic eye-screening program. Furthermore, we will investigate patients’, healthcare providers’, and policymakers’ perspectives on extension of annual screening intervals and establish a continuous feedback system. Additionally, we will address image quality and assessment concerns, and integrate the models with relevant software systems and workflows e.g., to ensure prompt extraction of demographic and risk factor data required to prescribe appropriate screening intervals, scheduling rescreening appointments based on risk level, and tracing and recalling patients who do not attend re-screening appointments.

In conclusion, we detailed a comprehensive protocol to develop and validate biennial and triennial screening models for DR and DME patients with T2DM using well characterized personal and clinical data, and progression probabilities in a large cohort of multi-ethnic Asians. Our preliminary finding that DR progression rates in primary care patients with T2DM were extremely low suggests that extending the screening interval beyond 12 months may be viable and safe, meaning that people at low-risk could be seen less frequently, freeing capacity to increase screening frequency of those at higher risk. Notwithstanding our promising preliminary results, our longer-term follow-up dataset is needed to confirm our recommendations to prolong screening intervals.

### Supplementary Information

**Supplementary Material 1. **

**Supplementary Material 2. **

## Data Availability

The datasets used and/or analysed during the current study are available from the corresponding author on reasonable request.
