# Supplementary material for: Extending the diabetic retinopathy screening intervals in Singapore: methodology and preliminary findings of a cohort study
Source: BMC Public Health. 2024 Mar 13;24:786. doi: 10.1186/s12889-024-18287-2 (PMC10935797; doi:10.1186/s12889-024-18287-2)
Supplement: Supplementary file 1 — Supplementary Material 1. [file 12889_2024_18287_MOESM1_ESM.docx]

**Recruitment**

Each polyclinic provides primary healthcare services to individuals staying in and around the region. In order to ensure a representative sample of Malays and Indians, Bedok and Pasir Ris SHPs were chosen to be part of the study as they receive a high attendance of these two minority groups. In addition, Outram and Bedok SHPs were included to maximize recruitment potential as they have high patient volume.

**Grading protocol**

All images were reviewed by a Level 1 trained primary reader and those showing signs of diabetic retinopathy were forwarded to a Level II more‐skilled secondary reader for confirmation. Audit checks of the human technician assessments were conducted regularly, and results showed 90% sensitivity (ability to correctly identify patients with a disease) and specificity (ability to correctly identify people without the disease) when measured against the “gold standard” of evaluation.

**Study assessments and data extraction**

On the day of baseline assessment, demographic (age, gender, ethnicity, marital status), socio-economic (education, family income, housing, occupation) and lifestyle (smoking, alcohol consumption) information was obtained via face-to-face, interviewer-administered questionnaires. Height (centimeters) and weight (kilograms) were obtained from the participant’s medical records. BP was assessed using a digital automatic BP monitor^36^ (Dinamap model Pro Series DP110X-RW, 100V2; GE Medical Systems Information Technologies, Inc., USA) if not available in the case notes. Participants’ HbA1c, lipids [low/high density lipoprotein cholesterol (LDL/HDL), triglycerides (TG), total cholesterol (TC)], serum creatinine, urine spot albumin: creatinine ratio (ACR), and estimated glomerular filtration rate (eGFR) were collected from the case notes if these parameters were assessed ≤12 months ago. Otherwise, a venous blood sample drawn via venepuncture and a mid-stream urine sample were collected and sent to Quest Laboratories Pte Ltd (Singapore) for same-day analysis. Distance VA, duration of DM, and medical history and medication were collected from participants’ polyclinic records.

**Data Quality Assurance and Control**

All study CRCs are trained by qualified personnel and are required to demonstrate competency in the relevant study procedures before being certified to perform them on participants. In addition, data collected and extracted are thoroughly checked by the CRCs to ensure that all data is complete and valid, before submitting for data entry.

**Data storage**

Data are collected in digital format via the Research Electronic Data Capture software (REDCap, Singapore). Clinical examination records, questionnaire responses, and biochemistry results are compiled into participant-specific case report forms that are labeled with the participant’s unique study number. Identifiable data is password-protected and stored in shared drives at each study site. Every quarter, data are extracted and checked for quality issues by the Data Management Unit at the Singapore Eye Research Institute.
