# Supplementary material for: Extending the diabetic retinopathy screening intervals in Singapore: methodology and preliminary findings of a cohort study
Source: BMC Public Health. 2024 Mar 13;24:786. doi: 10.1186/s12889-024-18287-2 (PMC10935797; doi:10.1186/s12889-024-18287-2)
Supplement: Supplementary file 2 — Supplementary Material 2. [file 12889_2024_18287_MOESM2_ESM.docx]

| **Supplementary Table 1. Grading levels of the Singapore integrated Diabetic Retinopathy Programme** | | | | |
| --- | --- | --- | --- | --- |
| Classification | Category | Approximate ETDRS equivalent | Description/features | Referral |
| **No DR** | None | Level 10 | - Normal Fundus - Any FH, CWS in the absence of DH/MA - Any HE in the absence of DH/MA (outside of outer zone) | Annual Rescreen |
| **NPDR** | Mild | Level 20-35 | One or more of the following:   - DH/MA - 1 or 2 BH† - HE in the absence of DH/MA (within macula zone)‡ | Annual Rescreen |
|  | Moderate | Level 43: moderate NPDR less than 4:2:1  Level 47: moderate NPDR less than 4:2:1 | One or more of the following:   - ≥ 3 BH§ - Any FH, CWS, HE with presence of DH/MA¶ - Venous beading in 1 quadrant | 3 months referral |
|  | Severe | 53A-E: severe to very severe NPDR, 4:2:1  rule | One or more of the following:   - 4 quadrants with each 10 or more hemorrhages (BH/DH/MA) - > 2 quadrants of venous beading - Any IRMA | 1 month referral |
| **PDR** | PDR | Levels 61,65,71,75,81,85: PDR, high-risk PDR, very severe or advanced PDR | One or more of the following:   - Any NVE - Any NVD - Tractional membranes/ pre-retinal fibrosis - Vitreous hemorrhage or pre-retinal hemorrhage | 1 week referral |
| Shaded columns in grey indicate participants with non-referable DR who were eligible for DRESS.  † Revised to <= 4 BH in August 2020  ‡ Revised to any FH, CWS, HE with presence of DH/MA in August 2020  § Revised to > 5 BH in August 2020  ¶ Revised to >=3 CWS, >=5 FH with presence of DH/MA in August 2020  *NPDR - Non-Proliferative Diabetic Retinopathy*  *PDR - Proliferative Diabetic Retinopathy; HMA - Hemorrhages and Microaneurysms; BH – Blot Hemorrhage; FH – Flame Hemorrhage; CWS - Cotton Wool Spots; HE - Hard Exudates; DH – Dot Hemorrhage; MA – Microaneurysms; IRMA – Intra-Retinal Microvascular Abnormality; NVE – Neovascularization Elsewhere; NVD -* Neovascularization of the Disc | | | | |

| **Supplementary Table 2. Classification, categorization, definition and referral recommendation for Diabetic Macular Edema** | | |
| --- | --- | --- |
| Classification | Definition | Referral |
| **No DME** | - No DR signs within the macula | Annual Rescreen |
| - **DME** | - Any MA/DH/BH within outer zone with VA better than 6/12 | Annual Rescreen |
|  | - Any MA/DH/BH within inner zone with VA better than 6/12†   Any HE within outer zone with VA better than 6/12 | 6 months Rescreen |
|  | - Any HE within outer zone‡   Any MA/DH/BH within outer zone with VA 6/12 or worse | 3 months referral |
|  | - Any MA/DH/BH within inner zone radius of fovea with VA 6/12 or worse - Any HE within inner zone | 1 month referral |
| Shaded cells in grey indicate participants with non-referable yearly rescreen DME who were eligible for DRESS.  † Revised to include any HE within outer zone with VA better than 6/12 in August 2020  ‡ Revised to any HE within outer zone with VA 6/12 or worse in August 2020  *DR - Diabetic retinopathy; DME – Diabetic Macular edema; MA – Microaneurysms; DH – Dot Hemorrhage; BH – Blot Hemorrhage; VA – Visual Acuity; HE - Hard Exudates* | | |

| **Supplementary Table 3. Baseline characteristics of Type 2 diabetes patients by age groups (n=4570)** | | | |  |
| --- | --- | --- | --- | --- |
| Characteristics | <55 years  (n = 1101) | 55-64 years  (n = 1867) | > 65 years  (n=1602) |  |
| Age (years), median (IQR) | 49.0 (44.0-52.0) | 60.0 (57.0-62.0) | 69.0 (67.0-72.0) |  |
| Male gender, n (%) | 592 (53.8) | 1000 (53.6) | 907 (56.6) |  |
| Diabetes duration (years), median (IQR) | 4.0 (2.0-7.0) | 6.0 (3.0-10.0) | 8.0 (4.0-14.0) |  |
| Ethnicity, n (%) |  |  |  |  |
| Chinese | 686 (62.3) | 1207 (64.6) | 1214 (75.8) |  |
| Malay | 272 (24.7) | 469 (25.1) | 259 (16.2) |  |
| Indian | 137 (12.4) | 181 (9.7) | 122 (7.6) |  |
| Eurasian | 6 (0.5) | 10 (0.5) | 7 (0.4) |  |
| Educational attainment, n (%) |  |  |  |  |
| Primary or lower | 124 (11.3) | 387 (20.7) | 523 (32.6) |  |
| Secondary or above | 977 (88.7) | 1479 (79.2) | 1079 (67.4) |  |
| Housing type, n (%) |  |  |  |  |
| Public | 983 (89.3) | 1657 (88.8) | 1394 (87.0) |  |
| Private | 112 (10.2) | 209 (11.2) | 206 (12.9) |  |
| Monthly Household Income, n (%) |  |  |  |  |
| < $2000 | 159 (14.4) | 412 (22.1) | 627 (39.1) |  |
| $2000 and above | 698 (63.4) | 947 (50.7) | 489 (30.5) |  |
| Occupation, n (%) |  |  |  |  |
| Unemployed | 52 (4.7) | 82 (4.4) | 28 (1.7) |  |
| Housewife | 118 (10.7) | 236 (12.6) | 158 (9.9) |  |
| Retired | 14 (1.3) | 288 (15.4) | 837 (52.2) |  |
| Working | 917 (83.3) | 1261 (67.5) | 579 (36.1) |  |
| Marital status, n (%) |  |  |  |  |
| Single or never married | 248 (22.5) | 195 (10.4) | 135 (8.4) |  |
| Married | 776 (70.5) | 1460 (78.2) | 1210 (75.5) |  |
| Separated, divorced or widowed | 77 (7.0) | 212 (11.4) | 257 (16.0) |  |
| Polyclinic Location, n (%) |  |  |  |  |
| Bedok | 327 (29.7) | 536 (28.7) | 377 (23.5) |  |
| Bukit Merah | 241 (21.9) | 347 (18.6) | 346 (21.6) |  |
| Outram | 202 (18.3) | 401 (21.5) | 548 (34.2) |  |
| Pasir Ris | 331 (30.1) | 583 (31.2) | 331 (20.7) |  |
| Lives alone (yes), n (%) | 57 (5.2) | 165 (8.8) | 157 (9.8) |  |
| Smoking status, n (%) |  |  |  |  |
| Never | 778 (70.7) | 1458 (78.1) | 1261 (78.7) |  |
| Past | 100 (9.1) | 185 (9.9) | 198 (12.4) |  |
| Current | 223 (20.3) | 224 (12.0) | 143 (8.9) |  |
| Alcohol use, n (%) |  |  |  |  |
| Never | 822 (74.7) | 1428 (76.5) | 1236 (77.2) |  |
| Past | 73 (6.6) | 109 (5.8) | 138 (8.6) |  |
| Current | 206 (18.7) | 330 (17.7) | 228 (14.2) |  |
| BMI (kg/m2), median (IQR) | 28.9 (25.6-33.1) | 26.7 (24.2-30.0) | 25.7 (23.4-28.3) |  |
| BMI categories (kg/m2) |  |  |  |  |
| < 18.5 | 4 (0.4) | 17 (0.9) | 14 (0.9) |  |
| 18.5 - 23.0 | 105 (9.5) | 250 (13.4) | 318 (19.9) |  |
| 23.0 - 27.5 | 323 (29.3) | 791 (42.4) | 749 (46.8) |  |
| > 27.5 | 662 (60.1) | 796 (42.6) | 516 (32.2) |  |
| Blood pressure (mmHg), median (IQR) |  |  |  |  |
| Systolic | 129.0 (120.0-137.0) | 130.0 (121.0-137.0) | 132.0(124.0-139.0) |  |
| Diastolic | 73.0 (67.0-80.0) | 71.0 (65.0-78.0) | 68.0 (62.5-74.0) |  |
| Hypertension^, n (%) | 713 (64.8) | 1473 (78.9) | 1435 (89.6) |  |
| Dyslipidaemia*, n (%) | 973 (88.4) | 1751 (93.8) | 1515 (94.6) |  |
| History of cardiovascular disease, n (%) |  |  |  |  |
| Coronary artery disease (yes) | 76 (6.9) | 200 (10.7) | 251 (15.7) |  |
| Stroke | 27 (2.5) | 65 (3.5) | 97 (6.1) |  |
| Kidney disease, n (%) | 82 (7.4) | 214 (11.5) | 343 (21.4) |  |
| Diabetes treatment, n (%) |  |  |  |  |
| Insulin | 96 (8.7) | 135 (7.2) | 68 (4.2) |  |
| Oral anti-diabetic medications | 925 (84.0) | 1449 (77.6) | 1126 (70.3) |  |
| PVA (Better eye) |  |  |  |  |
| None (LogMAR < 0.3) | 1071 (97.3) | 1801 (96.5) | 1472 (91.9) |  |
| Mild (LogMAR > 0.3) | 30 (2.7) | 66 (3.5) | 129 (8.1) |  |
| HbA1c (%), median (IQR) | 7.0 (6.5-7.8) | 6.8 (6.3-7.4) | 6.7 (6.3-7.2) |  |
| Lipids, median (IQR) |  |  |  |  |
| Total cholesterol (mmol/L) | 4.1 (3.6-4.8) | 4.1 (3.6-4.6) | 4.0 (3.5-4.4) |  |
| Triglycerides (mmol/L) | 1.4 (1.1-2.0) | 1.4 (1.0-1.9) | 1.3 (1.0-1.7) |  |
| HDL cholesterol (mmol/L) | 1.2 (1.0-1.4) | 1.3 (1.1-1.5) | 1.3 (1.1-1.6) |  |
| LDL cholesterol (mmol/L) | 2.2 (1.8-2.7) | 2.1 (1.7-2.5) | 2.0 (1.6-2.4) |  |
| Serum creatinine (umol/L), median (IQR) | 70.0 (58.0-82.0) | 74.0 (62.0-88.0) | 81.0 (67.0-95.2) |  |
| eGFR† (ml/min/1.72m2), median (IQR) | 101.8 (90.1-108.0) | 90.4 (77.3-97.1) | 76.4 (63.3-87.9) |  |
| Urine albumin/Creatinine (mg/mmol), median (IQR) | 2.0 (1.0-4.5) | 2.0 (1.1-4.5) | 2.2 (1.2-5.3) |  |
| BMI – Body Mass Index; DR – Diabetic Retinopathy; NPDR – Non-proliferative diabetic retinopathy; PVA – Presenting Visual Acuity; HbA1c – Haemoglobin A1c; IQR – Interquartlie range; HDL – High Density Lipoprotein; LDL – Low Density Lipoprotein; eGFR – Estimated Glomerular Filtration Rate  †eGFR was calculated based on the CKD-EPI formula; ^Hypertension – primary or secondary clinical diagnosis of hypertension in the medical records; *Dyslipidemia – primary or secondary clinical diagnosis of dyslipidemia in the medical records | | | |  |

| **Supplementary Table 4. Baseline characteristics of Type 2 diabetes patients by diabetes duration (n=4570)** | | | |  |
| --- | --- | --- | --- | --- |
| Characteristics | <5 years  (n = 1835) | > 5 years  (n = 2734) |  |  |
| Age (years), median (IQR) | 58.0 (52.0-64.0) | 63.0 (57.0-68.0) |  |  |
| Male gender, n (%) | 1012 (55.1) | 1486 (54.4) |  |  |
| Diabetes duration (years), median (IQR) | 2.0 (1.0-3.0) | 10.0 (7.0-14.0) |  |  |
| Ethnicity, n (%) |  |  |  |  |
| Chinese | 1231 (67.1) | 1875 (68.6) |  |  |
| Malay | 441 (24.0) | 559 (20.4) |  |  |
| Indian | 153 (8.3) | 287 (10.5) |  |  |
| Eurasian | 10 (0.5) | 13 (0.5) |  |  |
| Educational attainment, n (%) |  |  |  |  |
| Primary or lower | 343 (18.7) | 690 (25.2) |  |  |
| Secondary or above | 1491 (81.3) | 2044 (74.8) |  |  |
| Housing type, n (%) |  |  |  |  |
| Public | 1609 (87.7) | 2424 (88.7) |  |  |
| Private | 221 (12.0) | 306 (11.2) |  |  |
| Monthly Household Income, n (%) |  |  |  |  |
| < $2000 | 416 (22.7) | 782 (28.6) |  |  |
| $2000 and above | 918 (50.0) | 1215 (44.4) |  |  |
| Occupation, n (%) |  |  |  |  |
| Unemployed | 65 (3.5) | 97 (3.5) |  |  |
| Housewife | 217 (11.8) | 295 (10.8) |  |  |
| Retired | 352 (19.2) | 787 (28.8) |  |  |
| Working | 1201 (65.4) | 1555 (56.9) |  |  |
| Marital status, n (%) |  |  |  |  |
| Single or never married | 276 (15.0) | 302 (11.0) |  |  |
| Married | 1354 (73.8) | 2091 (76.5) |  |  |
| Separated, divorced or widowed | 205 (11.2) | 341 (12.5) |  |  |
| Polyclinic Location, n (%) |  |  |  |  |
| Bedok | 509 (27.7) | 731 (26.7) |  |  |
| Bukit Merah | 350 (19.1) | 584 (21.4) |  |  |
| Outram | 432 (23.5) | 718 (26.3) |  |  |
| Pasir Ris | 544 (29.6) | 701 (25.6) |  |  |
| Lives alone (yes), n (%) | 163 (8.9) | 216 (7.9) |  |  |
| Smoking status, n (%) |  |  |  |  |
| Never | 1384 (75.4) | 2112 (77.2) |  |  |
| Past | 184 (10.0) | 299 (10.9) |  |  |
| Current | 267 (14.6) | 323 (11.8) |  |  |
| Alcohol use, n (%) |  |  |  |  |
| Never | 1410 (76.8) | 2075 (75.9) |  |  |
| Past | 115 (6.3) | 205 (7.5) |  |  |
| Current | 310 (16.9) | 454 (16.6) |  |  |
| BMI (kg/m2), median (IQR) | 27.3 (24.7-31.1) | 26.3 (23.8-29.6) |  |  |
| BMI categories (kg/m2) |  |  |  |  |
| < 18.5 | 13 (0.7) | 22 (0.8) |  |  |
| 18.5 - 23.0 | 216 (11.8) | 457 (16.7) |  |  |
| 23.0 - 27.5 | 707 (38.5) | 1156 (42.3) |  |  |
| > 27.5 | 885 (48.2) | 1089 (39.8) |  |  |
| Blood pressure (mmHg), median (IQR) |  |  |  |  |
| Systolic | 131.0 (122.5-139.0) | 130.0 (121.0-137.5) |  |  |
| Diastolic | 72.0 (66.0-78.0) | 69.5 (63.0-76.0) |  |  |
| Hypertension^, n (%) | 1303 (71.0) | 2317 (84.7) |  |  |
| Dyslipidaemia*, n (%) | 1630 (88.8) | 2608 (95.4) |  |  |
| History of cardiovascular disease, n (%) |  |  |  |  |
| Coronary artery disease (yes) | 188 (10.2) | 339 (12.4) |  |  |
| Stroke | 73 (4.0) | 116 (4.2) |  |  |
| Kidney disease, n (%) | 164 (8.9) | 475 (17.4) |  |  |
| Diabetes treatment, n (%) |  |  |  |  |
| Insulin | 47 (2.6) | 252 (9.2) |  |  |
| Oral anti-diabetic medications | 1120 (61.0) | 2379 (87.0) |  |  |
| PVA (Better eye) |  |  |  |  |
| None (LogMAR < 0.3) | 1757 (95.7) | 2586 (94.6) |  |  |
| Mild (LogMAR > 0.3) | 78 (4.3) | 147 (5.4) |  |  |
| HbA1c (%), median (IQR) | 6.6 (6.2-7.2) | 6.9 (6.4-7.5) |  |  |
| Lipids, median (IQR) |  |  |  |  |
| Total cholesterol (mmol/L) | 4.3 (3.7-4.9) | 3.9 (3.5-4.4) |  |  |
| Triglycerides (mmol/L) | 1.4 (1.1-1.9) | 1.3 (1.0-1.8) |  |  |
| HDL cholesterol (mmol/L) | 1.3 (1.1-1.5) | 1.3 (1.1-1.5) |  |  |
| LDL cholesterol (mmol/L) | 2.2 (1.8-2.8) | 1.9 (1.6-2.3) |  |  |
| Serum creatinine (umol/L), median (IQR) | 73.0 (61.0-87.0) | 77.0 (63.0-91.0) |  |  |
| eGFR† (ml/min/1.72m2), median (IQR) | 91.0 (78.2-100.6) | 85.8 (70.3-95.8) |  |  |
| Urine albumin/Creatinine (mg/mmol), median (IQR) | 1.8 (1.0-3.8) | 2.2 (1.2-5.4) |  |  |
| BMI – Body Mass Index; DR – Diabetic Retinopathy; NPDR – Non-proliferative diabetic retinopathy; PVA – Presenting Visual Acuity; HbA1c – Haemoglobin A1c; IQR – Interquartlie range; HDL – High Density Lipoprotein; LDL – Low Density Lipoprotein; eGFR – Estimated Glomerular Filtration Rate  †eGFR was calculated based on the CKD-EPI formula; ^Hypertension – primary or secondary clinical diagnosis of hypertension in the medical records; *Dyslipidemia – primary or secondary clinical diagnosis of dyslipidemia in the medical records | | | |  |

| **Supplementary Table 5. Baseline characteristics of Type 2 diabetes patients by gender (n=4570)** | | | |  |
| --- | --- | --- | --- | --- |
| Characteristics | Male  (n =2499) | Female  (n = 2071) |  |  |
| Age (years), median (IQR) | 61.0 (55.0-68.0) | 61.0 (55.0-66.0) |  |  |
| Diabetes duration (years), median (IQR) | 6.0 (2.0-10.0) | 6.0 (3.0-10.0) |  |  |
| Ethnicity, n (%) |  |  |  |  |
| Chinese | 1806 (72.3) | 1301 (62.8) |  |  |
| Malay | 434 (17.4) | 566 (27.3) |  |  |
| Indian | 247 (9.9) | 193 (9.3) |  |  |
| Eurasian | 12 (0.5) | 11 (0.5) |  |  |
| Educational attainment, n (%) |  |  |  |  |
| Primary or lower | 476 (19.0) | 558 (26.9) |  |  |
| Secondary or above | 2022 (80.9) | 1513 (73.1) |  |  |
| Housing type, n (%) |  |  |  |  |
| Public | 2158 (86.4) | 1876 (90.6) |  |  |
| Private | 335 (13.4) | 192 (9.3) |  |  |
| Monthly Household Income, n (%) |  |  |  |  |
| < $2000 | 619 (24.8) | 579 (28.0) |  |  |
| $2000 and above | 1212 (48.5) | 922 (44.5) |  |  |
| Occupation, n (%) |  |  |  |  |
| Unemployed | 94 (3.8) | 68 (3.3) |  |  |
| Housewife | 2 (0.1) | 510 (24.6) |  |  |
| Retired | 668 (26.7) | 471 (22.7) |  |  |
| Working | 1735 (69.4) | 1022 (49.3) |  |  |
| Marital status, n (%) |  |  |  |  |
| Single or never married | 284 (11.4) | 294 (14.2) |  |  |
| Married | 2002 (80.1) | 1444 (69.7) |  |  |
| Separated, divorced or widowed | 213 (8.5) | 333 (16.1) |  |  |
| Polyclinic Location, n (%) |  |  |  |  |
| Bedok | 602 (24.1) | 638 (30.8) |  |  |
| Bukit Merah | 558 (22.3) | 376 (18.2) |  |  |
| Outram | 628 (25.1) | 523 (25.3) |  |  |
| Pasir Ris | 711 (28.5) | 534 (25.8) |  |  |
| Lives alone (yes), n (%) | 192 (7.7) | 187 (9.0) |  |  |
| Smoking status, n (%) |  |  |  |  |
| Never | 1527 (61.1) | 1970 (95.1) |  |  |
| Past | 455 (18.2) | 28 (1.4) |  |  |
| Current | 517 (20.7) | 73 (3.5) |  |  |
| Alcohol use, n (%) |  |  |  |  |
| Never | 1596 (63.9) | 1890 (91.3) |  |  |
| Past | 283 (11.3) | 37 (1.8) |  |  |
| Current | 620 (24.8) | 144 (7.0) |  |  |
| BMI (kg/m2), median (IQR) | 26.5 (24.1-29.5) | 27.1 (24.1-30.9) |  |  |
| BMI categories (kg/m2) |  |  |  |  |
| < 18.5 | 12 (0.5) | 23 (1.1) |  |  |
| 18.5 - 23.0 | 344 (13.8) | 329 (15.9) |  |  |
| 23.0 - 27.5 | 1128 (45.1) | 735 (35.5) |  |  |
| > 27.5 | 1000 (40.0) | 974 (47.0) |  |  |
| Blood pressure (mmHg), median (IQR) |  |  |  |  |
| Systolic | 130.5 (122.0-138.0) | 130.0 (121.0-138.0) |  |  |
| Diastolic | 71.0 (66.0-78.0) | 69.0 (63.0-76.0) |  |  |
| Hypertension^, n (%) | 2017 (80.7) | 1604 (77.5) |  |  |
| Dyslipidaemia*, n (%) | 2325 (93.0) | 1914 (92.4) |  |  |
| History of cardiovascular disease, n (%) |  |  |  |  |
| Coronary artery disease (yes) | 415 (16.6) | 112 (5.4) |  |  |
| Stroke | 134 (5.4) | 55 (2.7) |  |  |
| Kidney disease, n (%) | 409 (16.4) | 230 (11.1) |  |  |
| Diabetes treatment, n (%) |  |  |  |  |
| Insulin | 171 (6.8) | 128 (6.2) |  |  |
| Oral anti-diabetic medications | 1942 (77.7) | 1558 (75.2) |  |  |
| PVA (Better eye) |  |  |  |  |
| None (LogMAR < 0.3) | 2389 (95.6) | 1955 (94.4) |  |  |
| Mild (LogMAR > 0.3) | 109 (4.4) | 116 (5.6) |  |  |
| HbA1c (%), median (IQR) | 6.8 (6.3-7.4) | 6.8 (6.4-7.4) |  |  |
| Lipids, median (IQR) |  |  |  |  |
| Total cholesterol (mmol/L) | 3.9 (3.4 -4.4) | 4.2 (3.8-4.7) |  |  |
| Triglycerides (mmol/L) | 1.4 (1.0-1.9) | 1.3 (1.0-1.8) |  |  |
| HDL cholesterol (mmol/L) | 1.2 (1.0-1.4) | 1.4 (1.2-1.6) |  |  |
| LDL cholesterol (mmol/L) | 2.0 (1.6-2.5) | 2.1 (1.7-2.5) |  |  |
| Serum creatinine (umol/L), median (IQR) | 85.0 (75.0-97.0) | 62.0 (55.0-71.0) |  |  |
| eGFR† (ml/min/1.72m2), median (IQR) | 84.8 (71.1-95.2) | 91.4 (77.1-100.2) |  |  |
| Urine albumin/Creatinine (mg/mmol), median (IQR) | 2.0 (1.0-4.7) | 2.1 (1.2-4.8) |  |  |
| BMI – Body Mass Index; DR – Diabetic Retinopathy; NPDR – Non-proliferative diabetic retinopathy; PVA – Presenting Visual Acuity; HbA1c – Haemoglobin A1c; IQR – Interquartlie range; HDL – High Density Lipoprotein; LDL – Low Density Lipoprotein; eGFR – Estimated Glomerular Filtration Rate  †eGFR was calculated based on the CKD-EPI formula; ^Hypertension – primary or secondary clinical diagnosis of hypertension in the medical records; *Dyslipidemia – primary or secondary clinical diagnosis of dyslipidemia in the medical records | | | |  |

| **Supplementary Table 6. Overview of missing data** | |
| --- | --- |
|  | Missing data  n=2,571 |
| Educational attainment | 1 (0%) |
| Housing type | 9 (0.4%) |
| Monthly Household Income | 1,238 (48.2%) |
| BMI | 25 (1.0%) |
| Diabetes duration | 1 (0%) |
| HbA1c (%) | 8 (0.3%) |
| Total cholesterol (mmol/L) | 4 (0.2%) |
| Triglycerides | 4 (0.2%) |
| HDL cholesterol (mmol/L) | 4 (0.2%) |
| LDL cholesterol | 43 (1.7%) |
| Serum creatinine (umol/L) | 9 (0.4%) |
| eGFR† (ml/min/1.72m2) | 9 (0.4%) |
| Urine albumin/Creatinine (mg/mmol) | 1,216 (47.3%) |
